# Enhanced immunovirological response in women compared to men after antiretroviral therapy initiation during acute and early HIV‐1 infection: results from a longitudinal study in the French ANRS Primo cohort

**DOI:** 10.1002/jia2.25485

**Published:** 2020-04-25

**Authors:** Sophie Novelli, Pierre Delobel, Olivier Bouchaud, Véronique Avettand‐Fenoel, Pascale Fialaire, André Cabié, Faouzi Souala, François Raffi, Pilartxo Catalan, Laurence Weiss, Laurence Meyer, Cécile Goujard

**Affiliations:** ^1^ Paris‐Saclay University, UVSQ Inserm, CESP, U1018 Le Kremlin‐Bicêtre France; ^2^ Department of Infectious and Tropical Diseases Toulouse University Hospital Toulouse France; ^3^ Department of Infectious and Tropical Diseases Avicenne Hospital Assistance Publique–Hôpitaux de Paris (AP‐HP) Bobigny France; ^4^ Institut Cochin – CNRS 8104 INSERM U1016 AP‐HP Laboratoire de Microbiologie clinique Hôpital Necker‐Enfants Malades Université Paris Descartes Paris France; ^5^ Department of Infectious and Tropical Diseases Angers University Hospital Angers France; ^6^ Department of Infectious Diseases University Hospital of Martinique Fort‐de‐France France; ^7^ Department of Infectious and Tropical Diseases Rennes University Hospital Rennes France; ^8^ Infectious diseases department and Inserm CIC 1413 University Hospital of Nantes Nantes France; ^9^ Department of Internal Medicine AP‐HP Bicêtre Hospital Le Kremlin‐Bicêtre France; ^10^ Service d'Immunologie Clinique AP‐HP Hôpital Européen Georges Pompidou Paris France; ^11^ Inserm CESP U1018 Department of Public Health and Epidemiology AP‐HP Bicêtre Hospital Paris-Saclay University Le Kremlin‐Bicêtre France; ^12^ Inserm CESP U1018 Department of Internal Medicine AP‐HP Bicêtre Hospital Paris-Saclay University Le Kremlin‐Bicêtre France

**Keywords:** HIV infections, women’s health, treatment outcome, CD4:CD8 ratio, CD4 lymphocyte count, viral reservoir

## Abstract

**Introduction:**

Previous studies have reported better immunovirological characteristics in women compared with men after HIV seroconversion. We investigated whether differences persisted under long‐term antiretroviral therapy (ART) in individuals treated since acute and early HIV‐1 infection (AHI).

**Methods:**

Data were obtained for 262 women and 1783 men enrolled between 1996 and 2017 in the French multicentre ANRS PRIMO cohort. We modelled the viral response, long‐term immune recovery and HIV DNA decay in the 143 women and 1126 men who initiated ART within the first three months of infection.

**Results:**

The participants were mostly white. The mean age was 37 years at AHI diagnosis. Pre‐ART viral loads were lower in women than men, 5.2 and 5.6 log_10_ copies/mL (*p = *0.001). After ART initiation, women more rapidly achieved viral suppression than men (adjusted hazard ratio: 1.33, 95% confidence interval 1.09 to 1.69). They also experienced a faster increase in CD4^+^ T‐cell count and CD4:CD8 ratio during the first months of treatment. Sex‐related differences in CD4^+^ T‐cell counts were more pronounced with increasing age. This led to a sustained mean difference of 99 to 168 CD4^+^ T‐cells/µL depending on age between women and men at 150 months of ART. Moreover, CD4:CD8 ratio of women was higher than that of men by 0.31, at 150 months of ART. There was no statistically significant difference between sexes for the levels of HIV DNA over time (mean estimate at the last modelling point: 1.9 log_10_ copies/10^6^ PBMCs after 70 months of ART for both sexes).

**Conclusions:**

The high level of immune recovery and decrease in total HIV DNA levels achieved after ART initiation during AHI reinforce the importance of early diagnosis of HIV infection and immediate ART initiation. The immunological benefit of being female increased throughout prolonged ART duration, which may give women additional protection from adverse clinical events and premature ageing.

## 1 Introduction

Women comprise more than half of people living with HIV worldwide and have been disproportionately affected by HIV in many regions, in particular young women aged 15 to 24 years [1]. Significant sex differences have been described in the course of HIV‐1 disease. Most studies in treatment‐naïve patients diagnosed with acute or chronic HIV‐1 infection have shown that women have two‐ to six‐fold lower viral loads than men, after adjusting for potential confounders [2]. The mechanisms that mediate this difference are still not fully understood. Hormones and genes contribute significantly to sex‐related differences in outcomes of infection [3]. Differences in the innate function of human plasmacytoid dendritic cells (pDCs) have been established, with pDCs from women exhibiting enhanced TLR7‐mediated IFN‐α production than pDCs from men [4]. Moreover, both oestrogen and progesterone have been reported to modulate pDCs IFN‐α secretion (reviewed by [5, 6]).

Determinants of health inequalities other than gender, such as socioeconomic status and geographic origin, may confound comparison of HIV outcomes between the sexes [7]. Women have shown a lower risk of both mortality and AIDS‐related mortality than men in a setting with free access to healthcare and reduced gender inequalities [8]. The effectiveness of antiretroviral therapy (ART) in terms of CD4^+^ T‐cell recovery and viral suppression appears to be generally comparable across sexes in chronic infection [6, 9]. Nevertheless, research on acute and early HIV infection (hereafter referred to as AHI (acute and early HIV‐1 infection)) tends to be preferentially carried out on men who have sex with men [10], and few studies have assessed sex‐related disparities in treatment outcomes when ART was initiated during AHI. A previous study reported similar rates of virological suppression and CD4^+^ T‐cell gains between men and women [11]. Finally, recent reviews have highlighted the lack of data on sex‐related differences in the establishment and size of the viral reservoir [6, 12]. Previous studies have described lower total HIV DNA in women than in men in the first months after infection [13] and under long‐term suppressive ART [14, 15, 16] but no study has assessed it longitudinally.

In France, women accounted for one third of people living with HIV (PLHIV) and 35% of new HIV diagnoses in 2018 [17]. We thus aimed to longitudinally assess whether differences reported in early infection between the sexes persist after years under ART, for women enrolled during AHI in the French National Agency for Research on AIDS and Viral Hepatitis (ANRS) CO6 PRIMO cohort. We modelled the immunovirological response of 143 women treated from AHI with ART over 12 years, and compared their outcomes with those of men.

## 2 Methods

### 2.1 Study participants

Since 1996 the ANRS PRIMO Cohort has enrolled in 95 French hospitals patients presenting with acute or early HIV‐1 infection, according to the following criteria: (1) an incomplete western blot (i.e. absence of anti‐p68 and/or anti‐p34), (2) detectable p24 antigenaemia or detectable plasma viral load, associated with either a negative or weakly reactive enzyme‐linked immunosorbent assay (ELISA) or a negative western blot or (3) an interval <3 months between a negative and positive ELISA. The cohort was approved by the Ile‐de‐France‐3 Ethics Committee and patients gave their informed consent to participate.

### 2.2 Measurements

CD4^+^ T‐cell counts and plasma HIV‐1 RNA were routinely measured at enrolment, at months 1, 3 and 6 and every six months thereafter. Plasma and whole blood samples were collected and frozen at enrolment, then at months 1, 3, 6 and 12, and every 12 months thereafter. Total cell‐associated HIV‐1‐DNA was measured at a central laboratory from frozen peripheral blood mononuclear cells (PBMCs), using the real‐time PCR GENERIC HIV‐DNA assay, with a threshold of <20 copies/10^6^ PBMCs [18]. Fiebig staging was estimated afterwards for the analyses from data collected at enrolment in the cohort.

### 2.3 Statistical analyses

All participants who were enrolled in the ANRS PRIMO cohort within the period between June 1996 through November 2017, and with a viral load and a CD4^+^ T‐cell count measurement prior to ART initiation were eligible for this study.

First we analysed sex‐related differences in immunovirological markers during AHI using multiple linear regression models, adjusted for potential confounders including age and geographical origin (Sub‐Saharan African vs. others). Continuous variables that did not fulfil the linearity assumption were modelled using fractional polynomials.

Then we focused on early treated participants, that is participants who initiated ART within the first three months of infection. The date of infection was estimated as the date of symptom onset minus 15 days, or the date of the incomplete western blot finding minus one month or the midpoint between a negative and a positive ELISA result for asymptomatic patients. Among these early‐treated patients, we estimated the time to viral suppression and the time to viral rebound under ART after the first viral suppression. We defined the time to viral suppression as the time to the first viral load <50 copies/mL after starting ART. Time to viral rebound was defined as the time to the first single viral load >1000 copies/mL or the first of two consecutive viral loads >500 copies/mL after achievement of viral suppression within the 12 months following ART initiation. Hazard ratios (HRs) for the association between sex and time to viral response/rebound were calculated using Cox proportional hazards models, which allowed us to adjust for potential cofounders. Finally, we modelled and compared the dynamics of CD4^+^ T‐cell counts, CD4:CD8 ratios and HIV DNA levels under ART between the sexes, using piecewise linear mixed‐effects models with random coefficients. Follow‐up was truncated at 150 months for the mixed models (70 months for HIV DNA), given that the subsequent measures represented less than 5% of the studied subjects. To estimate the magnitude of sex‐related differences by increasing age, we regressed predicted CD4^+^ counts after 150 months of ART on sex, age and an interaction term between age and sex. Some participants had experienced planned ART interruptions in clinical trials. Such individuals were censored on the date of ART discontinuation for interruptions ≥15 days for the modelling of viral response and viral rebound, and ≥3 months otherwise. All analyses were performed using R software (R Core Team 2015).

## 3 Results

### 3.1 Study population

We studied 262 women and 1783 men enrolled in the PRIMO cohort (Figure S1). The mean age at AHI diagnosis was 37 years. Participants were mostly (95%) infected by the sexual route. Fiebig stages at AHI diagnosis were 36.3, 41.8 and 20.5% of stages I to III, IV and V to VI respectively (1.4% missing data).

We compared the baseline characteristics of the female and male participants (Table 1). Median age (interquartile range, IQR) was 36 (28 to 46) and 36 (29 to 44) years for women and men respectively. Women experienced symptomatic AHI less often, and had an average of 49 more CD4^+^ T‐cells/µL than men (*p = *0.002), along with lower plasma viral loads and HIV DNA levels (mean differences: −0.5 log_10_ copies/mL, *p < *0.0001 and −0.1 log_10_ copies/10^6^ PBMCs, *p = *0.003 respectively). These sex‐related differences persisted after adjusting for potential confounders, including age, geographical origin, smoking status, time from infection and calendar period. Alternative adjustment for Fiebig stages instead of time from infection led to similar results.

**Table 1:** Characteristics of patients at the acute and early HIV‐1 infection diagnosis in the ANRS Primo cohort

Characteristics	Women (n = 262)	Men (n = 1783)	*p*‐value^a^
Age, years	38.0 (12.2)	37.0 (10.6)	0.12
Geographical origin, Sub‐Saharan African	19.1 (50)	3.3 (58)	<0.0001
Education level			<0.0001
Primary	13.4 (35)	6.6 (117)	
Secondary	52.3 (137)	37.1 (661)	
Tertiary	27.9 (73)	51.9 (926)	
Smoker	33.2 (87)	43.7 (779)	0.001
Route of HIV acquisition			0.45
Sexual route	94.3 (247)	94.9 (1692)	
Injecting drug use	0.4 (1)	0.2 (3)	
Other or Unknown	5.3 (14)	4.9 (88)	
Positive CMV serology^b^	76.0 (199)	79.8 (1423)	0.03
Hepatitis B co‐infection^c^	4.5 (11)	2.5 (41)	0.08
Hepatitis C co‐infection^d^	2.8 (7)	3.0 (48)	0.88
Fiebig stages			0.32
I	0 (0)	0 (0)	
II to III	31.3 (82)	37.1 (661)	
IV	45.0 (118)	41.3 (736)	
V	10.7 (28)	9.8 (175)	
VI	11.8 (31)	10.4 (185)	
Missing	1.1 (3)	1.4 (26)	
B Subtype	36.6 (96)	67.9 (1211)	<0.0001
Symptomatic acute HIV‐1 infection	80.5 (211)	88.4 (1576)	<0.0001
CD4^+^ T‐cell count, cells/μL	584 (255)	535 (247)	0.002
CD4:CD8 ratio	0.7 (0.5)	0.6 (0.4)	<0.0001
Plasma HIV RNA, log_10_ copies/mL	4.8 (1.2)	5.3 (1.1)	<0.0001
HIV DNA level, log_10_ copies/10^6^ PBMCs	3.2 (0.6)	3.3 (0.6)	0.003
Time from infection to ART initiation			0.02
≤3 months	54.6 (143)	63.2 (1126)	
>3 months	36.6 (96)	30.5 (543)	
Did not initiate ART	8.8 (23)	6.4 (114)	

Data are presented as the mean (standard deviation) or percentage (n). ART, antiretroviral therapy; CMV, cytomegalovirus; ANRS, National Agency for Research on AIDS and Viral Hepatitis; HIV, human immunodeficiency virus; PBMCs, peripheral mononuclear blood cells.

^a^ Z‐test for continuous data, χ^2^ or Fisher’s exact test for categorical data

^b^ data available for 232 women and 1574 men

^c^ participants were classified as HBV co‐infected if positive for HbS antigenaemia. Data available for 247 women and 1632 men

^d^ participants were classified as HCV co‐infected if HCV‐positive by PCR or serology test. Data available for 247 women and 1595 men.

**Figure 1:**
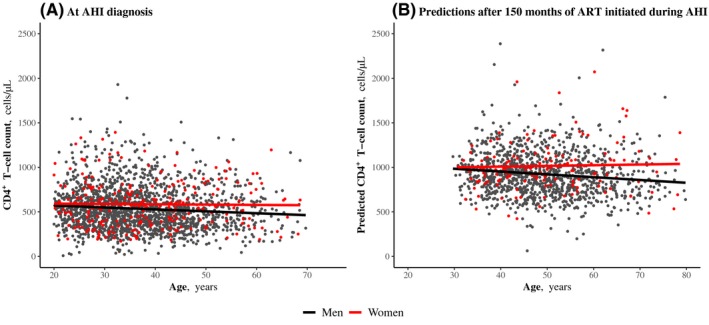
Sex‐related differences in CD4^+^ T‐cell count depending on age. **(a)** At AHI diagnosis. Points over 2500 cells/µL were cut from the graph for better visibility. **(b)** After 150 months of ART initiated during AHI. Scatterplot of predicted values of CD4^+^ T‐cell count obtained by mixed model. Regression lines of predicted values on age, depending on sex. Points over 2500 cells/µL were cut from the graph for better visibility. AHI, acute and early HIV infection; ART, antiretroviral therapy.

We found evidence of an interaction of age with sex‐related differences in CD4^+^ T‐cell counts and HIV DNA levels measured at AHI diagnosis: CD4^+^ T‐cell counts decreased and HIV DNA levels increased with age in men, whereas these parameters remained stable in women. Thus, sex‐related differences were more marked in older participants (Figure 1a, Table S1).

The median follow‐up in the cohort was 66.1 months (95% confidence interval (CI), 63.5 to 71.5), estimated using the reverse Kaplan‐Meier method. Retention in the cohort was similar between the sexes: the adjusted (a)HR of being lost to follow‐up or voluntarily interrupting the follow‐up was of 0.99, 95% CI (0.79 to 1.23) for women vs. men (Table 2).

**Table 2:** Retention in care in the PRIMO cohort and virological outcomes after ART initiation during acute HIV‐1 infection according to sex

Outcomes	Univariate analysis		Multivariate analysis	
	HR (95% CI)	*p*‐value	HR (95% CI)	*p*‐value
**All participants**				
Withdrawal of follow‐up^a^				
Men, n = 1679	–	–	–	–
Women, n = 239	0.99 (0.79, 1.23)	0.91	0.98 (0.78, 1.23)	0.89
**Patients who initiated ART within three months of infection**				
Viral suppression below 50 copies/mL^b^				
Men, n = 896	–	–	–	–
Women, n = 109	1.28 (1.06, 1.55)	0.01	1.33 (1.09, 1.69)	0.03
Viral suppression below 50 copies/mL^c^ ART initiation after 2008				
Men, n = 596	–	–	–	–
Women, n = 52	1.43 (1.10, 1.86)	0.01	1.88 (1.34, 2.65)	<0.0001
Viral rebound under ART^d^				
Men, n = 833	–	–	–	–
Women, n = 105	1.59 (0.75, 3.40)	0.23	1.19 (0.55, 2.56)	0.66

Results are based on Cox proportional hazards models. ART, antiretroviral therapy; HR, hazard ratio.

^a^ Loss to follow‐up or voluntary cessation. The multivariate model was adjusted for age, geographical origin and education level

^b^ the multivariate model was adjusted for age, geographical origin, time from infection to ART initiation and body mass index and stratified by quantiles of viral load at ART initiation and year of ART initiation

^c^ same as b, plus the multivariate model was stratified by first ART regimen (with or without integrase inhibitor)

^d^ first single viral load >1000 copies/mL or the first of two consecutive viral loads >500 copies/mL after achievement of viral suppression within 12 months following ART initiation. The multivariate model was adjusted for age and geographical origin and stratified by calendar period.

Sixty women experienced pregnancy and 45 gave birth during their follow‐up in the cohort. Clinical AIDS‐events were rare during the follow‐up period: three women and 45 men developed AIDS‐defining events. All‐cause mortality did not differ between the sexes (log rank test *p = *0.73). Nine deaths (3.4%) occurred among women, of which one was AIDS‐related (pneumonia), and 44 (2.5%) among men, of which four were AIDS‐related (tuberculosis, 2; non‐Hodgkin lymphoma, 2). Most of the participants who died (88%) had a last viral load measurement <50 copies/mL (measurement within the year before death).

### 3.2 Responses to ART after initiation during AHI

We further focused on the 143 women and 1126 men who initiated ART within three months of infection. The first line ART regimen was based on a boosted protease inhibitor (PI) for most individuals. Among women, 45% received a boosted PI‐based regimen, 20% an integrase inhibitor (INSTI)‐based regimen and 11% a non‐nucleoside reverse transcriptase inhibitors (NNRTI)‐based regimen. Among men, the distribution was 53, 27 and 6% for PI‐, INSTI‐ and NNRTI‐based regimens respectively.

Mean pre‐ART HIV RNA levels were 5.2 and 5.6 log_10_ copies/mL for women and men respectively (*p = *0.001). Rates of viral suppression after ART initiation were high; women demonstrated a shorter time to viral response than men (log rank test, *p* = 0.01). By six months, 72.3% of women (95% CI, 63.0 to 79.2) and 61.3% of men (95% CI, 58.2 to 64.2) achieved viral suppression at a threshold of <50 copies/mL. By 12 months, this frequency had increased to 94.3% of women (95% CI, 87.7 to 97.4) and 88.3% of men (95% CI, 86.0 to 90.2). Women still showed higher rate of viral suppression than men in a Cox model that took into account other predictive factors, including time from infection and pre‐ART viral load: adjusted hazard ratio (aHR) 1.33, 95% CI (1.09 to 1.69), *p = *0.03 (Table 2). This better response in women was even more marked when considering only participants enrolled after 2008, mostly treated with more potent drugs such as darunavir/ritonavir and integrase inhibitors: aHR 1.88, 95% CI (1.34 to 2.65), *p < *0.0001. Viral rebound under ART after a first viral response occurred in 5% of the participants, with no difference in time to rebound between sexes.

**Figure 2:**
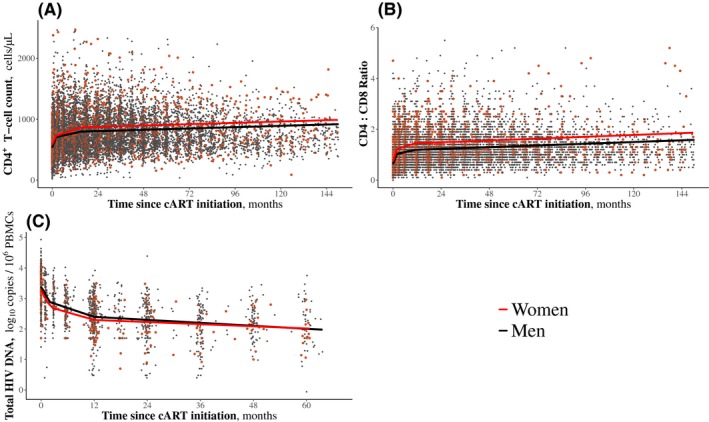
Immunovirological marker dynamics after ART initiation during acute and early HIV infection. Immunovirological marker dynamics were estimated using mixed models with random coefficients. **(a)** CD4^+^ T‐cell count: model run from 142 women and 1113 men. Points over 2000 cells/µL were cut from the graph for better visibility. **(b)** CD4:CD8 ratio: model run from 142 women and 1110 men. Points over 6 were cut from the graph for better visibility. **(c)** HIV DNA levels: model run from 70 women and 353 men. ART, antiretroviral therapy.

Next we longitudinally modelled CD4^+^ T‐cell recovery, CD4:CD8 ratio restoration and HIV DNA decay under ART. The three markers followed a classical three‐slope dynamics, with all slopes significantly differing from 0. Increases in the CD4^+^ T‐cell count and CD4:CD8 ratio and decline in HIV DNA levels were most marked during the first two months of ART and gradually slowed down thereafter, but continued through the end of follow‐up (Figure 2). Overall, participants achieved high levels of immune restoration and reduction of total HIV DNA levels. After 150 months of ART, the mean (95% CI) CD4^+^ T‐cell count and CD4:CD8 ratio were 923 cells/µL (883 to 963) and 1.61 (1.5 to 1.7), and the mean HIV DNA level after 70 months of ART was 1.9 log_10_ copies/10^6^ PBMCs (1.8 to 2.0).

We assessed specific long‐term sex‐related effects through models adjusted for pre‐ART viral load, time from infection to ART initiation, geographical origin, age at ART initiation and calendar period (Table S2). At ART initiation, women had higher CD4^+^ T‐cell counts than men, and this was even more marked in older individuals in the cohort, as previously shown for all participants in AHI (Figure 1a). In addition to such a pre‐ART advantage, women experienced a faster increase in CD4^+^ T‐cell counts than men during the first months of ART (+3.4 cells/µL per month from month 2 to month 15, *p = *0.03). The difference between men and women thus strengthened on ART. For example women who were 45 years old at ART initiation had a mean of 100 cells/µL more than men of the same age (*p *< 0.0001) and after 150 months of ART, the difference was 151 cells/µL (*p = *0.02) (Table 3, Figure 1b).

**Table 3:** Mean estimated difference in immunovirological marker levels between the sexes

Age at ART initiation, years	At ART initiation		After 150 months under ART	
	Estimate (95% CI)	*p*‐value^a^	Estimate (95% CI)	*p*‐value^a^
Difference in CD4^+^ T‐cells count, women versus men (reference), cells/µL^b^				
30	+ 48 (−1, 95)	0.05	+ 99 (−34, 231)	0.14
35	+ 65 (22, 107)	0.002	+ 116 (−13, 246)	0.09
40	+ 83 (40, 123)	<0.0001	+133 (4, 263)	0.04
45	+ 100 (52, 145)	<0.0001	+151 (20, 281)	0.02
50	+ 118 (63, 172)	<0.0001	+168 (35, 301)	0.01

	At ART initiation		After 150 months under ART	
	Estimate (95% CI)	*p*‐value^a^	Estimate (95% CI)	*p*‐value^a^
Difference in the CD4:CD8 Ratio, women versus men (reference)^c^				
Regardless of age at ART initiation	+0.12 (0.04, 0.19)	0.003	+0.31 (0.03, 0.60)	0.03

Age at ART initiation, years	At ART initiation		After 70 months under ART	
	Estimate (95% CI)	*p*‐value^a^	Estimate (95% CI)	*p*‐value^a^
Difference in HIV DNA levels, women versus men (reference), log_10_ copies/10^6^ PBMCs^d^				
30	−0.01 (−0.17, 0.16)	0.95	+0.12 (−0.18, 0.43)	0.43
35	−0.07 (−0.21, 0.07)	0.33	+0.06 (−0.24, 0.35)	0.71
40	−0.14 (−0.28, 0.004)	0.06	−0.10 (−0.31, 0.29)	0.95
45	−0.20 (−0.36, −0.05)	0.01	−0.07 (−0.38, 0.23)	0.62
50	−0.27 (−0.46, −0.08)	0.01	−0.14 (−0.46, 0.18)	0.38

Estimates were predicted by a mixed model with random coefficients. ART, antiretroviral therapy

^a^ Significance test of the difference from 0

^b^ the model was adjusted for geographical origin, age at antiretroviral therapy (ART) initiation, viral load at ART initiation, time from infection to ART initiation, smoking status as a time‐dependent variable and calendar period

^c^ the model was adjusted for geographical origin, age at ART initiation, viral load at ART initiation, time from infection to ART initiation, smoking status as a time‐dependent variable, calendar period and CMV coinfection status at baseline

^d^ the model was adjusted for geographical origin, age at ART initiation, viral load at ART, time from infection to ART initiation and calendar period.

Similarly, women had a higher mean CD4:CD8 ratio than men at ART initiation (+0.12 in mean, *p = *0.003). Women experienced a faster increase in their CD4:CD8 ratio during the first two months of treatment, leading to an even more marked mean difference of +0.31 after 150 months of ART (*p = *0.03). Among participants treated for more than three years, 77% of women and 66% of men had a CD4:CD8 ratio >1 at their last visit.

Women over the age of 40 years had lower HIV DNA levels than men at ART initiation, whereas there was no significant sex‐related difference in younger participants. With long‐term ART, women and men achieved similar levels of HIV DNA, regardless of their age at ART initiation (Table 3).

### 3.3 Sensitivity analyses

For all the analyses, an alternative adjustment for viral subtype instead of geographical origin only marginally changed the estimations of sex‐related differences. Moreover, models either adjusted for viral subtype and restricted to non‐African participants, or adjusted for the geographical origin and restricted to participants infected by a non‐B subtype, led to results consistent with the main analysis. Finally, exclusion of women who experienced pregnancy during follow‐up only marginally changed the results.

## 4 Discussion

We assessed sex‐related disparities in HIV outcomes for 262 women and 1783 men of the French ANRS PRIMO Cohort, one of the largest cohorts of subjects diagnosed during AHI. At AHI diagnosis, women had better immunovirological parameters, that is significantly higher CD4^+^ T‐cell counts and CD4:CD8 ratios and lower viral loads than men, as already described in seroconverters [11, 19, 20]. We assessed how these sex‐related differences evolved after ART initiation during AHI over a period of more than 12 years of ART. Women experienced a faster viral suppression than men, along with a faster increase in their CD4^+^ T‐cell counts and CD4:CD8 ratios during the first months of treatment, leading to even more marked differences between men and women for these parameters. After 150 months of ART, women had a mean of +99 to 168 more CD4^+^ T‐cells/µL than men and CD4:CD8 ratio of women was higher than that of men by 0.31. Both women and men achieved low levels of HIV DNA: 1.9 log_10_ copies/10^6^ PBMCs in mean after 70 months of ART, with no sex‐related difference.

A major strength of our study was the long period of follow‐up, up to 12.5 years from AHI diagnosis. Moreover, we could adjust for most known confounders apart from age, such as smoking status, geographical origin, viral subtype and cytomegalovirus status. Taking into account geographical origin was of particular interest since some recent studies showed an association between ethnicity and reservoir size [21, 22]. We focused on participants treated since their AHI diagnosis because they received management close to current recommendations [23, 24]*.*

As in other studies [25, 26, 27], ART initiation during AHI rapidly led to high rates of viral suppression and near 90% of participants were virally suppressed by 12 months. Subsequent viral rebound while on ART was uncommon and time to viral rebound was similar between men and women. The better viral response we observed in women was not explained by differences in pre‐ART viral loads or first ART regimen and contrasts with the results of most previous studies, including subgroup analyses of recent trials, which found similar rates of virological suppression between the sexes [9, 28, 29, 30, 31, 32]. However, these studies were conducted with participants who mostly initiated ART during later stages of infection. One cohort study of individuals diagnosed and mainly treated from AHI reported no sex‐related difference in virological response, but at a threshold of 400 copies/mL, and in a context of socioeconomic conditions specific to the USA, as quoted by the authors [11]. This is certainly another strength of our study, in which we compared virological responses in men and women with a threshold of 50 copies/mL and in a large number of women.

Both men and women achieved high levels of immune restoration. The dynamics of CD4^+^ T‐cell counts, CD4:CD8 ratios and HIV DNA levels were still ongoing at the end of follow‐up. This confirms the benefit of early ART initiation and long‐term ART to sustainably improve immune recovery [33, 34, 35, 36]. The immunological advantage women had over men during AHI strengthened over the first year of ART and was then maintained with sustained ART. Some studies have reported that women have greater CD4^+^ gain than men one to three years after initiating ART [31, 37, 38, 39, 40, 41], whereas in others, CD4^+^ T‐cell counts rose similarly between men and women [29, 42, 43, 44, 45]. Here we observed that women retained a higher CD4^+^ T‐cell count than men throughout follow‐up under ART. After 12.5 years, women had a mean of 99 to 168 more CD4^+^ cells/µL than men. We also observed a long‐term difference of +0.31 in the CD4:CD8 ratio between women and men, in accordance with the results of previous studies [34, 46, 47, 48, 49]. Such differences in CD4^+^ T‐cell counts and CD4:CD8 ratio are similar to that reported between the sexes in non‐HIV individuals [50, 51, 52]. Higher CD4 counts and CD4:CD8 ration observed in women under long‐term ART compared to men may be partially or totally explained by a return to sex‐related differences observed in uninfected individuals. ART may restore the normal homeostatic regulation of CD4^+^ T‐cell numbers by suppressing HIV‐1 replication [40]. A low CD4:CD8 ratio has been linked to increased risk of non‐AIDS morbidity, independently of CD4^+^ T‐cell count [53, 54]. The CD4:CD8 ratio generally negatively correlates with markers of immune activation [55]. Thus, the difference in the CD4^+^ T‐cell count and the CD4:CD8 ratio we observed may be of clinical relevance from a life‐long perspective.

We did not observe any sex‐related difference in long‐term levels of total HIV DNA under ART, contrary to two cross‐sectional studies that reported lower levels in women [14, 15]. Most of participants of these studies were chronic initiators, whereas ART initiated during AHI is a major determinant of viral reservoir reduction [16, 56, 57, 58]. Here, HIV persistence was evaluated by measure of total HIV DNA in peripheral blood. This marker has been shown to be a clinical relevant marker [59] and a good predictor of the viral outgrowth [60]. Nevertheless, it does not reflect HIV reservoir activity, which may differ between the sexes. In a limited number of participants who were matched for the duration of viral suppression, CD4^+^ T‐cell count and nadir CD4^+^ T‐cell count, Scully *et al* [61] reported that despite similar HIV DNA levels in CD4^+^ T‐cells, women showed lower levels of HIV reservoir activity than men, estimated by both single copy assay and multiply spliced HIV RNA. Interestingly, another cross‐sectional study reported a non‐statistically significant trend toward a smaller replication‐competent reservoir in women compared with men in Ugandan virally suppressed participants, but not in Americans [22]. Such differences in HIV reservoir activity between the sexes but also ethnic groups are relevant to strategies for an HIV cure and need to be evaluated in larger cohorts.

Sex‐related differences in CD4^+^ T‐cell counts were more pronounced with increasing age. We observed that the CD4^+^ T‐cell count decreased with age in men, both at AHI diagnosis and after years of sustained ART, whereas it did not in women. This adds to results of a previous study, in which the better short‐term CD4^+^ responses in women, with respect to men, tended to be more pronounced among individuals ≥50 years of age (vs <50) [38]. The reasons for such a differential impact of age according to sex in ageing patients is yet to be clarified, including the effect of the menopause status in HIV women. Decreased levels of INF‐α in women that go along with low oestrogen levels after the menopause could play a role by sustaining CD4^+^ T‐cell count in older women. IFN‐α contributes to innate control of infection even if it may also provide target cells for the virus during acute infection and in that way be deleterious for the outcome of infection. Higher levels of IFN‐α in younger women might lead to increased target cell recruitment to HIV replication sites and thus explained similar levels of HIV DNA levels between men and women in younger participants.

Our study has some limitations. Data on adherence were not available. Nevertheless, the large majority of participants showed a sustained virological response, similar between men and women. Therefore, the long‐term immune differences in response to ART that we observed between the sexes are unlikely to be explained by differences in adherence to treatment. Another limit may be the underrepresentation of women included in the PRIMO cohort compared to the population of PLHIV in France. Thirteen per cent of our participants were women, whereas women represent 23% of all early HIV diagnosis in France in 2018 [17]. Moreover, more regular screening of HIV infection in men who have sex with men explain why they were more likely to be diagnosed with AHI and therefore enrolled in the PRIMO cohort than women. The underrepresentation of women in HIV clinical research is still an important caveat and needs to be addressed worldwide in clinical settings.

## 5 ConclusionS

Our results show the high immunological efficacy of long‐term ART in men and women and its sustainability. The immunological benefit of being female increased throughout prolonged ART duration. Whether this increased benefit may give women additional protection from adverse clinical events and premature ageing is yet to be further investigated.

## Competing interests

The authors declare that they have no competing interests.

## Authors’ contributions

LM and CG designed the cohort and the research study. AC, FR, FS, LW, OB, PC, PD, PF and VA‐F, contributed the data. SN performed the statistical analyses and LM supervised them. SN, LM and CG wrote the paper. All authors read and approved the final manuscript.

## Abbreviations

aHR, adjusted hazard ratio; AHI, acute and early HIV‐1 infection; ANRS, National Agency for Research on AIDS and Viral Hepatitis; ART, antiretroviral therapy; ELISA, enzyme‐linked immunosorbent assay; HR, hazard ratio; PBMCs, peripheral mononuclear blood cells; pDCs, plasmacytoid dendritic cells; PLHIV, people living with HIV.

## Supporting information


**Appendix S1.** Members of the ANRS PRIMO cohort.
**Figure S1.** Eligible participants of the ANRS PRIMO cohort.
**Table S1.** Sex and age effects on markers measured during acute and early HIV infection in 1783 men and 262 women in the ANRS PRIMO cohort
**Table S2.** Immunovirological marker dynamics after antiretroviral therapy initiation during acute and early HIV infection
