# Supplementary material for: Enhanced immunovirological response in women compared to men after antiretroviral therapy initiation during acute and early HIV‐1 infection: results from a longitudinal study in the French ANRS Primo cohort
Source: J Int AIDS Soc. 2020 Apr 25;23(4):e25485. doi: 10.1002/jia2.25485 (PMC7183251; doi:10.1002/jia2.25485)
Supplement: Supplementary file 1 — Appendix S1. Members of the ANRS PRIMO cohort. Figure S1. Eligible participants of the ANRS PRIMO cohort. Table S1. Sex and age effects on markers measured during acute and early HIV infection in 1783 men and 262 women in the ANRS PRIMO cohort Table S2. Immunovirological marker dynamics after antiretroviral therapy initiation during acute and early HIV infection [file JIA2-23-e25485-s001.docx]

# Additional files

## Supplementary Figure 1. Eligible participants of the ANRS PRIMO cohort


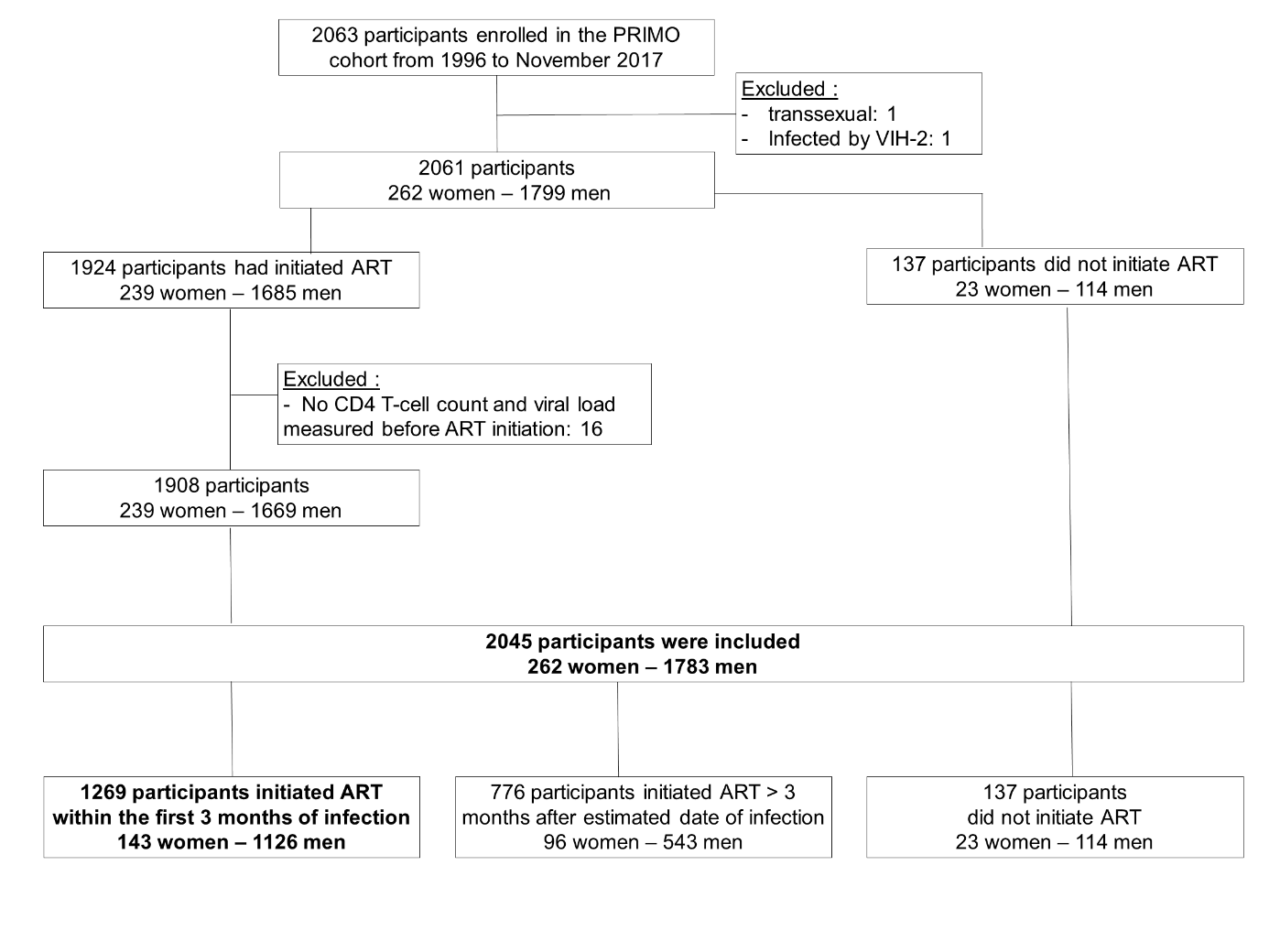


## Supplementary Table 1. Sex and age-related effects on markers measured during ****acute and early HIV infection**** in 1783 men and 262 women in the ANRS PRIMO cohort

| **Baseline predictor** | **Univariate analysis** | | |  | **Multivariate analysis** | | |
| --- | --- | --- | --- | --- | --- | --- | --- |
|  | **Estimate (95%CI)** | **P-value** | |  | **Estimate (95%CI)** | **P-value** | |
| **A. HIV RNA levels,** log_10_ copies /mL ^a^ | | | | | | |  |
| **Sex** |  |  | |  |  |  | |
| **Men** | Reference | - | |  | - | - | |
| **Women** | -0.43 (-0.57, 0.30) | < 0.0001 | |  | -0.34 (-0.47, -0.21) | < 0.0001 | |
|  |  |  | |  |  |  | |
| **Per a 10-year positive difference in age** ^†^ | 0.09 (0.05, 0.14) | < 0.0001 | |  | 0.08 (0.05, 0.12) | < 0.0001 | |
|  |  |  | |  |  |  | |
| **B. CD4^+^ T-cell count,** cells/μL ^b^ | | | | | | |  |
| **Sex** | not shown because the sex-related difference depends on age due to the significant interaction of age on the association between sex and CD4^+^ T-cell count | | | | | |  |
|  |  | | | | | |  |
| **Per 10-year increase in age** ^‡^ | |  | |  |  |  | |
| **Men** | -18.3 (-29.2, -7.4) | 0.001 | |  | -12.6 (-23.9, -1.9) | 0.02 | |
| **Women** | -2.6 (-27.2, 22.1) | 0.84 | |  | 1.6 (-22.4, 25.6) | 0.89 | |
|  |  |  | |  |  |  | |
| **C. HIV DNA levels,** log_10_ copies /10^6^ PBMCs ^c^ | | | | | | |  |
| **Sex** | not shown because the sex-related difference depends on age due to the significant interaction of age on the association between sex and HIV DNA levels | | | | | |  |
|  |  | | | | | |  |
| **Per 10-year increase in age** ^‡^ | |  | |  |  |  | |
| **Men** | 0.07 (0.04, 0.10) | < 0.0001 | |  | 0.07 (0.04, 0.10) | < 0.0001 | |
| **Women** | -0.02 (-0.09, 0.05) | 0.56 | |  | -0.01 (-0.08, 0.05) | 0.72 | |
|  |  |  | |  |  |  | |
| **D. CD4:CD8 Ratio** ^d^ |  |  | |  |  |  | |
| **Sex** |  |  | |  |  |  | |
| **Men** | Reference | - | |  | - | - | |
| **Women** | 0.13 (0.08, 0.19) | < 0.0001 | |  | 0.16 (0.10, 0.23) | < 0.0001 | |
|  |  |  | |  |  |  | |
| **Per 10-year positive difference in age** ^†^ | 0.001 (-0.002, 0.002) | | 0.95 |  | 0.01 (-0.01, 0.03) | 0.31 | |
| ^†^Age effect did not differ between sexes; one coefficient is shown.  ^‡^ Age effect significantly differed between sexes; thus we showed two coefficients corresponding to men- and women-specific age effects  ^a^ The multivariate model was adjusted for geographical origin, time from infection, and calendar period, and run on 1749 men and 252 women.  ^b^ Same as a, plus the multivariate model was also adjusted for smoking status. The multivariate model was run on 1681 men and 245 women.  ^c^ Same as a. The multivariate model was run on 1442 men and 202 women.  ^d^ Same as a, plus the multivariate model was also adjusted for smoking status and cytomegalovirus serology. The multivariate model was run on 1480 men and 222 women. | | | | | | |  |

## ****Supplementary Table 2. Immunovirological marker dynamics after**** antiretroviral therapy ****initiation during acute and early HIV infection****

| **A. CD4^+^ T-cell count,** **mean gain per month** (cells/µL/month) | | | | | | |
| --- | --- | --- | --- | --- | --- | --- |
| **Period** | **Univariate estimate**  **(standard error)** | **P-value** | |  | **Adjusted estimate**  **(standard error)** | **P-value** |
| **0 – 2 months** |  |  | |  |  |  |
| **Men** | Reference | **-** | |  | **-** | **-** |
| **Women** | -13.6 (10.7) | 0.21 | |  | -6.4 (10.7) | 0.55 |
|  |  |  | |  |  |  |
| **2 – 15 months** |  |  | |  |  |  |
| **Men** | Reference | **-** | |  | **-** | **-** |
| **Women** | +3.9 (1.6) | 0.01 | |  | +3.4 (1.6) | 0.03 |
|  |  |  | |  |  |  |
| **15 – 150 months** |  |  | |  |  |  |
| **Men** | Reference | **-** | |  | **-** | **-** |
| **Women** | +0.01 ( 0.5) | 0.98 | |  | +0.04 (0.48) | 0.94 |
| Individuals were censored when ART cessation > 3 months.  The multivariate model was adjusted for geographical origin, age at antiretroviral therapy (ART) initiation, viral load at ART initiation, time from infection to ART initiation, smoking status as a time-dependent variable and calendar period. | | | | | | |
|  | | | | | | |
| **B. CD4:CD8 ratio, mean gain per month** | | | | | | |
| **Period** | **Univariate estimate**  **(standard error)** | **P-value** | |  | **Adjusted estimate ^a^**  **(standard error)** | **P-value** |
| **0 – 2 months** |  |  | |  |  |  |
| **Men** | Reference | **-** | |  | - | - |
| **Women** | +0.05 (0.02) | 0.02 | |  | +0.06 (0.02) | 0.01 |
|  |  |  | |  |  |  |
| **2 – 11 months** |  |  | |  |  |  |
| **Men** | Reference | **-** | |  | - | - |
| **Women** | +0.007 (0.004) | 0.11 | |  | +0.003 (0.004) | 0.48 |
|  |  |  | |  |  |  |
| **11 – 150 months** |  |  | |  |  |  |
| **Men** | Reference | **-** | |  | - | - |
| **Women** | +0.0002 (0.001) | 0.85 | |  | +0.0003 (0.001) | 0.77 |
| Individuals were censored when ART cessation > 3 months.  The multivariate model was adjusted for geographical origin, age at ART initiation, viral load at ART initiation, time from infection to ART initiation, smoking status as a time-dependent variable, calendar period and status for CMV coinfection at baseline | | | | | | |
|  | | | | | | |
| **C- HIV-DNA levels**, **mean gain per month** (log_10_ copies/10^6^ PBMCs) | | | | | | |
| **Period** | **Univariate estimate**  **(standard error)** | **P-value** |  | | **Adjusted estimate**  **(standard error)** | **P-value** |
| **0 – 2 months** |  |  |  | |  |  |
| **Men** | Reference | **-** |  | | **-** | **-** |
| **Women** | -0.03 (0.05) | 0.55 |  | | -0.04 (0.05) | 0.45 |
|  |  |  |  | |  |  |
| **2 – 12 months** |  |  |  | |  |  |
| **Men** | Reference | **-** |  | | **-** | **-** |
| **Women** | +0.01(0.01) | 0.55 |  | | +0.01 (0.01) | 0.51 |
|  |  |  |  | |  |  |
| **12 – 75 months** |  |  |  | |  |  |
| **Men** | Reference | **-** |  | | **-** | **-** |
| **Women** | +0.002 (0.003) | 0.82 |  | | +0.002 (0.003) | 0.36 |
| Individuals were censored when ART cessation > 3 months.  The multivariate model was adjusted for geographical origin, age at ART initiation, viral load at ART initiation, time from infection to ART initiation and calendar period. | | | | | | |

## Appendix

**Members of the ANRS PRIMO are as follows:**

- Thierry ALLEGRE, Centre hospitalier général d’Aix en Provence, Service d’Hématologie
- Djamila MAKHLOUFI, Jean-Michel LIVROZET, François JEANBLANC, Pierre CHIARELLO, Valérie ARTIZZU, Hôpital Edouard Herriot de Lyon, Immunologie Clinique
- Patrick MIAILHES, Joseph KOFFI, Valérie THOIRAIN, Corinne BROCHIER, Sophie PAILHES, Lyon La Croix Rousse, Services d’Hépato-Gastroentérologie et des Maladies Infectieuses
- Anne FRESARD, Elisabeth BOTELHO-NEVERS, Amandine GAGNEUX-BRUNON, Véronique RONAT, CHU Saint Etienne, Service des Maladies Infectieuses et Tropicales
- Alain LAFEUILLADE, Gisèle PHILIP, Gilles HITTINGER, Assi ASSI, Véronique LAMBRY, Hôpital Font-Pré de Toulon, Médecine Interne, Hémato-Infectiologie

-  Eric ROSENTHAL, Alissa NAQVI, Brigitte DUNAIS, Eric CUA, Christian PRADIER, Jacques DURANT, Sophie BREAUD, Hôpital L’Archet, Nice, Service de Médecine Interne, Maladies Infectieuses et Tropicales

- Denis QUINSAT, Serge TEMPESTA, Centre Hospitalier d’Antibes, Service de Médecine Interne
- Isabelle RAVAUX, Hôpital de la Conception de Marseille, Service des Maladies Infectieuses
- Isabelle POIZOT MARTIN, Olivia FAUCHER, Hélène LAROCHE, Hôpital Sainte Marguerite de Marseille, Unité d'Hématologie
- Hélène CHAMPAGNE, Emilie RACAMIER, Centre Hospitalier de Valence, Maladies Infectieuses et Tropicales
- Gilles PICHANCOURT, Centre Hospitalier Henri Duffaut d’Avignon, Service Hématologie Maladies Infectieuses
- Philippe MORLAT, Thierry PISTONE, Fabrice BONNET, Isabelle FAURE, Mojgan HESSAMFAR, Denis LACOSTE, Marie-Carmen PERTUSA, Marie-Anne VANDENHENDE, Noëlle BERNARD, François PACCALIN, Cédric MARTELL, Marie-Catherine RECEVEUR, Pierre DUFFAU, Denis DONDIA, Emmanuel RIBEIRO, Sabrina CALTADO, Hôpital Saint André de Bordeaux, Médecine Interne.
- Didier NEAU, Michel DUPONT; Hervé DUTRONC, Frédéric DAUCHY, Charles CAZANAVE, Thierry PISTONE, Marc-Olivier VAREIL, Thierry PISTONE, Gaétane WIRTH, Séverine LE PUIL, Hôpital Pellegrin de Bordeaux, Maladies Infectieuses.
- Jean-Luc PELLEGRIN, Isabelle RAYMOND, Jean-François VIALLARD, Stéphanie DELAGE, Hôpital Haut Lévèque de Bordeaux, Médecine Interne et Maladies Infectieuses
- Daniel GARIPUY, Hôpital Joseph Ducuing de Toulouse, Médecine Interne
- Pierre DELOBEL, Martine OBADIA, Lise CUZIN, Muriel ALVAREZ, Noemie BIEZUNSKI, Lydie PORTE, Patrice MASSIP, Alexa DEBARD, Florence BALSARIN, Sandra LAGARRIGUE, Julie KELLER, Hôpital Purpan de Toulouse, SMIT-CISIH
- François PREVOTEAU DU CLARY, Christian AQUILINA, Cité de la santé Toulouse
- Jacques REYNES, Vincent BAILLAT, Corinne MERLE, Vincent LEMOING, Nadine ATOUI, Alain MAKINSON, Jean Marc JACQUET, Christina PSOMAS, Antoine VILLADERO, Christine TRAMONI, Hôpital Gui de Chauliac de Montpellier, Service des Maladies Infectieuses et Tropicales
- Hugues AUMAITRE, Mathieu SAADA, Marie MEDUS, Orélia EDEN, Ségolène NEUVILLE, Milagros FERREYRA, Laetitia PINHEIRO, Hôpital Saint Jean de Perpignan, Service des Maladies Infectieuses
- Albert SOTTO, Claudine BARBUAT, Isabelle ROUANET, Didier LEUREILLARD, Jean-Marc MAUBOUSSIN, Catherine LECHICHE, Régine DONSESCO, CHU de Nîmes-Caremeau, Service des Maladies Infectieuses et Tropicales.
- Valérie GABORIEAU, Gilles DUMONDIN, Laure FERRAN, CH de Pau, Service des Maladies Infectieuses et Tropicales.
- André CABIE, Sylvie ABEL, Sandrine PIERRE-FRANCOIS, Anne-Sophie BATALA, Christophe CERLAND, Régine DUPIN, Joanie BAVAY, CHU Fort de France, Hôpital de Jour

- Isabelle LAMAURY, Ketty SAMAR, CHU de Pointe à Pitre/ABYMES, Service de Dermatologie / Maladies Infectieuses

- Catherine GAUD, Carole RICAUD, Roland RODET, Guillaume WARTEL, Sandrine GAZAIGNES, Carmele SAUTRON, CHU de la Reunion, site Felix Guyon, Service d’Immunologie

- Geneviève BECK-WIRTH, Catherine MICHEL, Jean-Michel PETER, Charles BECK, Jean-Michel HALNA, Meryem BENOMAR, Groupe Hospitalier de la région de Mulhouse et Sud-Alsace, GHRMSA, UF Déficit Immunitaire
- Catherine CHIROUZE, Christine DROBACHEFF-THIEBAUT, Jean-François FAUCHER, Adeline FOLTZER, François PARCELIER, Catherine BOURDEAUX, Jean-Marie ESTAVOYER, Ozeka BABRE, Mathieu HUSTACHE, Hôpital St Jacques de Besançon, Service des Maladies Infectieuses et de Dermatologie
- Lionel PIROTH, Pascal CHAVANET, Michel DUONG, Marielle BUISSON, Sandrine TREUVELOT, Sandrine GOHIER, Carole CHARLES, Hôpital du Bocage de Dijon, Service des Maladies Infectieuses
- Bruno HOEN, Thierry MAY, Marie-Pierre BOUILLON, CHU de Vandoeuvre-lès-Nancy, Hôpital de Brabois, Service des Maladies Infectieuses et Tropicales
- Mahsa MOHSENI ZADEH, Martin MARTINOT, Anaïs MOTHES, Anne PACHART, Hôpital Louis Pasteur de Colmar, Service d’Immunologie Clinique
- Benoît MARTHA, Noëlle JEUNET, Centre Hospitalier William Morey de Chalon Sur Saône, Service de Médecine Interne

- David REY, Maria PARTISANI, Christine CHENEAU, Michèle PRIESTER, Claudine BERNARD-HENRY, Erick De MAUTORT, Patricia FISCHER, Service le Trait d’Union, Hôpitaux Universitaires de Strasbourg

- Jean-Luc BERGER, Isabelle KMIEC, Hôpital Robert Debré, Service des Maladies Infectieuses, Reims.

- Olivier ROBINEAU, Thomas HULEUX, Faïza AJANA, Isabelle ALCARAZ, Christophe ALLIENNE, Véronique BACLET, Agnès MEYBECK, Michel VALETTE, Nathalie VIGET, Christophe ALLIENNE, Emmanuelle AISSI, Raphael BIEKRE, Pauline CORNAVIN, Centre Hospitalier DRON de Tourcoing, Service de Maladies Infectieuses

- Moise MACHADO, Marie Lise LECAPITAINE, Jean-Christophe SEGHEZZI, Centre Hospitalier de Compiègne, Service de Médecine Interne

- Georges DIAB, C H de la Haute Vallée de l’Oise de Noyon, Service de Médecine

- François RAFFI, Bénédicte BONNET, Clotilde ALLAVENA, Olivier GROSSI, Véronique RELIQUET, Eric BILLAUD, Cecile BRUNET, Sabelline BOUCHEZ, Pascale MORINEAU-LE HOUSSINE, Fabienne SAUSER, David BOUTOILLE, Michel BESNIER, Morane CAVELLEC, Albane SORIA, Hôtel-Dieu de Nantes, CISIH Médecine Interne
- Christophe MICHAU, Carole GRAND-COURAULT, Centre Hospitalier de Saint Nazaire, Pôle Médecine polyvalente Adulte – Gériatrique
- Iuliana DARASTEANU, Françoise ITOUA, Sylvie APRELON, Centre Hospitalier de Chartres, Hôpital Louis Pasteur, Service des Maladies Infectieuses et Tropicales
- Thierry PRAZUCK, Laurent HOCQUELOUX, Barbara DE-DIEULEVEUT, Centre Hospitalier Régional d’Orléans, Hôpital de la Source, Service des Maladies Infectieuses
- Faouzi SOUALA, Christian MICHELET, Pierre TATTEVIN, Cédric ARVIEUX, Matthieu REVEST, Helene LEROY, Jean-Marc CHAPPLAIN, Matthieu DUPONT, Fabien FILY, Jocelyne VIVENT, CHRU Pontchaillou de Rennes, Clinique des Maladies Infectieuses
- Louis BERNARD, Frédéric BASTIDES, Olivier BOURGAULT, Hôpital Bretonneau de Tours, Service des maladies Infectieuses
- Renaud VERDON, Arnaud DE LA BLANCHARDIERE, Anne MARTIN, Philippe FERET, CH régional Côte de Nacre de Caen, Service de Maladies Infectieuses
- Loïk GEFFRAY, Hôpital Robert Bisson de Lisieux, Service de Médecine Interne
- Rodolphe BUZELE, Centre Hospitalier La Beauchée de Saint-Brieuc, Médecine Interne et Maladies Infectieuses
- Pascale FIALAIRE, Jean Marie CHENNEBAULT, Valérie RABIER, Pierre ABGUEGUEN, Sami REHAIEM, Centre Hospitalier Régional d’Angers, Service des Maladies Infectieuses
- Odile LUYCX, Philippe MOREAU, Centre Hospitalier Bretagne Sud de Lorient, Service d’Hématologie
- Yves POINSIGNON, Anabèle DOS SANTOS, Virginie MOUTON- RIOUX, Centre Hospitalier Bretagne Atlantique de Vannes, Service de Medecine Interne et Maladies Infectieuses
- Mourad MOUHADJER, Centre Hospitalier d’Alençon, Médecine 2
- Philippe PERRE,Sophie LEANTEZ-NAINVILLE, Jean-Luc ESNAULT, Thomas GUIMARD, Laetitia LAINE, Centre Hospitalier Départemental de La Roche sur Yon, Service de Médecine
- Jean-Jacques GIRARD, Véronique SIMONET, Hôpital de Lôches, Service de Médecine Interne
- Yasmine DEBAB, David THERON, CHU Charles Nicolle de Rouen, Maladies Infectieuses et Tropicales

- Christine JACOMET, Justine PROUTEAU, Hôpital Gabriel-Montpied de Clermont Ferrand, Service des Maladies Infectieuses et Tropicales

- Claire GENET, Hôpital DUPUYTREN de Limoges, Maladies Infectieuses et Tropicales

- Bruno ABRAHAM, Centre Hospitalier de Brive, Departement de maladies Infectieuses

- Florence GOURDON, Centre Hospitalier de Vichy, Service de Médecine Interne

- Odile ANTONIOTTI, Centre Hospitalier de Montluçon, Dermatologie

- Jean-Michel MOLINA, Caroline LASCOUX-COMBE, Samuel FERRET, Matthieu LAFAURIE, Nathalie COLIN DE VERDIERE, Diane PONSCARME, Nathalie DE CASTRO, Alexandre ASLAN, Willy ROZENBAUM, Claire PINTADO, François CLAVEL, Olivier TAULERA, Caroline GATEY, Anne-Lise MUNIER, Pauline PENOT, Guillaume CONORT, Nathalie LEROLLE, Anne LEPLATOIS, Stéphanie BALAUSINE, Jeannine DELGADO, Hôpital Saint Louis de Paris, Service des Maladies Infectieuses et Tropicales
- Anne-Sophie LASCAUX, Isabelle TURPAULT, Hôpital Saint Louis de Paris, Clinique MST
- Laurence GERARD, Hôpital Saint Louis de Paris, Service d'Immunologie Clinique
- Pierre-Marie GIRARD, Diane BOLLENS, Nadia VALIN, Pauline CAMPA, Benedicte LEFEBVRE, Muriel TOURNEUR, Laurent FONQUERNIE, Charlotte WEMMERT, Zineb OUAZENE, Jean-Luc LAGNEAU, Manuela LE CAM, Julie LAMARQUE, Hôpital Saint Antoine de Paris , Service des Maladies Infectieuses et Tropicales
- Yazdan YAZDANPANAH, Bao PHUNG, Adriana PINTO, Dorothée VALLOIS, Ornella CABRAS, Françoise LOUNI, Solaya CHALAL, G. Hospitalier Bichat-Claude Bernard de Paris, Service de Maladies Infectieuses et Tropicales
- Gilles PIALOUX, Thomas LYAVANC, Valérie BERREBI, Julie CHAS, Anne ADDA LIEVIN, Mouniya MEBARKI, Hopital Tenon de Paris, Service des Maladies Infectieuses
- Agathe RAMI, Myriam DIEMER, Maguy PARRINELLO, Hôpital Lariboisière de Paris, Service de Médecine Interne A
- Dominique SALMON, Loïc GUILLEVIN, Tassadit TAHI, Catherine CHAKVETADZE, Linda BELARBI, Olivier ZAK DIT ZBAR, Odile LAUNAY, Benjamin SILBERMANN, Firouze BANI SADR, Marie-Pierre PIETRI, G. H. Cochin de Paris, Département de Médecine Interne, Hôtel Dieu de Paris, Unité fonctionnelle de Pathologie Infectieuse
- Anne SIMON, Manuela BONMARCHAND, Naouel AMIRAT, François PICHON, Jean-Luc VOURCH, Myriam KIRSTETTER, Dalila BENIKEN, G. H. Pitié-Salpétrière de Paris, Service de Médecine Interne
- Christine KATLAMA, Marc Antoine VALANTIN, Roland TUBIANA, Fabienne CABY, Luminita SCHNEIDER, Sophie SEANG, Hind STITOU, Saadia BEN ABDALLAH, Ludovic LENCLUME, G. H. Pitié-Salpétrière de Paris, Service des Maladies Infectieuses
- Laurence WEISS, Martin BUISSON, Dominique BATISSE, Marina KARMOCHINE, Juliette PAVIE, Didier JAYLE, Philippe CASTEL, Jean DEROUINEAU, Pascale KOUSIGNAN, Murielle ELIAZEVITCH, Lio COLLIAS, Marie-Laure LUCAS, Hôpital Européen Georges Pompidou de Paris, Service d'Immunologie Clinique
- Jean-Paul VIARD, Jacques GILQUIN, Laurence SLAMA, Alain SOBEL, Jade GHOSN, Blanka HADACEK, Nugyen THU-HUYN, Agnes CROS, Marie-Josée DULUCQ, Hôtel Dieu de Paris, Centre de Diagnostic et Thérapeutique
- Paul Henri CONSIGNY, Claudine DUVIVIER, Fanny LANTERNIER, Fatima TOUAM, Carole LOUISIN, Centre Médical de l’Institut Pasteur de Paris, Service des Maladies Infectieuses
- Loïc BODARD, Françoise CHURAQUI, Institut Mutualiste Montsouris de Paris, Département de Médecine Interne
- Cécile GOUJARD, Yann QUERTAINMONT, Martin DURACINSKY, Olivier SEGERAL, Arnaud BLANC, Delphine PERETTI, Valérie SEGUIN, Antoine CHERET, Alicia DE CASTRO, Alain-Serge KEITA, Hôpital de Bicêtre, Médecine Interne
- Jean Daniel LELIEVRE, Yves LEVY, Anne Sophie LASCAUX, Chrystel CHESNEL, Raida BEN RAYANA, Hôpital Henri Mondor de Créteil, Immunologie Clinique
- François BOUE, Sophie ABGRALL, Réma BAYOUD, Hôpital Antoine Béclère de Clamart, Médecine Interne et Immunologie Clinique
- Pierre DE TRUCHIS, Morgane MARCOU, Hôpital Raymond Poincaré de Garches, Service des Maladies Infectieuses et Tropicales
- Alain DEVIDAS, Amélie CHAVROL, Solène PATRA-DELON, Nouara AGHER, Hôpital de Corbeil-Essonnes, Service Hématologie
- Olivier BOUCHAUD, Mohamed Kassim YOUSSOUF, Arezki BENMAMMAR, Hôpital Avicenne de Bobigny, Maladies Infectieuses et Tropicales
- Elisabeth ROUVEIX, Soumia CHENAKEB, Hôpital Ambroise Paré de Boulogne, Médecine Interne
- Alix GREDER BELAN, Claire GODIN COLLET, Perrine DAYLE, Audrey THERBY, Safia SOUAK, Dominique BORNAREL, Hôpital du Chesnay, CH Andre Mignot du Chesnay, Maladies Infectieuses et Tropicales
- Emmanuel MORTIER, Martine BLOCH, Anne-Marie SIMONPOLI, Véronique MANCERON, Isabelle CAHITTE, Emmanuel HIRAUX, Erik LAFON, François CORDONNIER, Ai-feng ZENG, Hôpital Louis Mourier de Colombes, Médecine Interne
- David ZUCMAN, Catherine MAJERHOLC, Amina FADLI, Hôpital Foch de Suresnes , Médecine Interne
- Agnès ULUDAG, Agnès. LEFORT, Christine BAZIN, Abdelmoula BECHAREF Hôpital Beaujon de Clichy, Médecine Interne
- Vincent DANELUZZI, Ghania BOUTERIA, Bénedicte MONTOYA, Centre Hospitalier de Nanterre, Service de Médecine Interne
- Julie BOTTERO, Flory MFUTILA KAYKAY, Arezki BENMAMMAR, Hôpital Jean Verdier de Bondy, Service de Médecine Interne, Unité de Maladies Infectieuses
- Olivier PATEY, Jonas BANTSIMBA, Sophie DELLLION, Pauline CARAUX PAZ, Laurent RICHIER, Centre Hospitalier Intercommunal de Villeneuve St Georges, Médecine Interne
- Valérie GARRAIT, Isabelle DELACROIX, Brigitte ELHARRAR, Laurent RICHIER, Centre Hospitalier Intercommunal de Créteil, Médecine Interne, Hépato-Gastroentérologie
- Daniel VITTECOQ, Claudine BOLLIOT, Hôpital de Bicêtre, Service de Maladies Infectieuses et Tropicales
- Annie LEPRETRE, Gersendre GRAIN, Hôpital Simone Veil d’Eaubonne, Médecine 2, Consultation ESCALE
- Philippe GENET, Virginie MASSE, Juliette GERBE, Consultation d’Immuno/Hématologie d’Argenteuil
- Laurent BLUM, Gersendre GRAIN, Hôpital René Dubos, Service de Dermatologie
- Véronique PERRONE, Centre Hospitalier François Quesnay de Mantes La Jolie, Service des Maladies Infectieuses
- Eka CHAKVETADZE, Hicham ROUKAS, Centre Hospitalier Marc Jacquet de Melun, Service de Médecine
- Eric FROGUEL, Phlippe SIMON, Sylvie TASSI, Centre Hospitalier de Marne-la-Vallée, Service de Médecine Interne
- Benoit CAZENAVE, Yves WELKER, Catherine LECLERC, Bénédicte MONTOYA, Centre Hospitalier Intercommunal Poissy-Saint Germain en Laye, Service des Maladies Infectieuses
